# Cardiovascular Complications in Patients with Heart Failure and COVID-19: CARDIO COVID 19-20 Registry

**DOI:** 10.3390/jcdd11020034

**Published:** 2024-01-23

**Authors:** Mario Speranza, Juan D. López-López, Pedro Schwartzmann, Igor Morr, María Juliana Rodríguez-González, Andrés Buitrago, Freddy Pow-Chon-Long, Luiz Carlos Passos, Víctor Rossel, Eduardo Roque Perna, Manuela Escalante, Alexander Romero, Andrea Alejandra Arteaga-Tobar, Daniel Quesada, Walter Alarco, Juan Esteban Gómez-Mesa

**Affiliations:** 1 Department of Cardiology, Hospital Clínica Bíblica, San José 10104, Costa Rica; sacagce@icloud.com; 2 Consejo Interamericano de Falla Cardiaca e Hipertensión Pulmonar (CIFACAH) de la Sociedad Interamericana de Cardiología (SIAC), Mexico City 01000, Mexico; dr.freddypowchl@gmail.com (F.P.-C.-L.); vrossel@uchile.cl (V.R.); pernaucic@hotmail.com (E.R.P.); alexrom2876@me.com (A.R.); daniel.quesada@acc.co.cr (D.Q.); walarco@hotmail.com (W.A.); 3 Fundación Valle del Lili, Centro de Investigaciones Clínicas, Cali 760026, Colombia; juan.lopez.lo@fvl.org.co (J.D.L.-L.); manuela.escalante@fvl.org.co (M.E.); andrea.arteaga@fvl.org.co (A.A.A.-T.); 4 Department of Health Sciences, Universidad Icesi, Cali 760031, Colombia; 5 Department of Cardiology, CAPED—Advanced Research Center and Hospital Unimed, Ribeirão Preto 14000-000, Brazil; pedrovs.usp@gmail.com; 6 Department of Tropical Cardiology, Sociedad Venezolana de Cardiología, Caracas 1060, Venezuela; igormorr@gmail.com; 7 Department of Cardiology, LaCardio-Fundación Cardio Infantil, Bogotá D.C. 110131, Colombia; mjrodriguez@cardioinfantil.org; 8 Department of Cardiology, Fundación Santa Fe, Bogotá D.C. 110111, Colombia; abuitrag@uniandes.edu.co; 9 Department of Cardiology, Hospital Luis Vernaza, Guayaquil 090313, Ecuador; 10 Department of Cardiology, Universidad Espíritu Santo, Guayaquil 092301, Ecuador; 11 Department of Cardiology, Hospital Ana Nery, Salvador 40323-900, Brazil; lcpassos@ufba.br; 12 Instituto Nacional del Tórax, Heart Transplant Program, Santiago de Chile 8320000, Chile; 13 Division of Heart Failure and Pulmonary Hypertension, Instituto de Cardiología J.F. Cabral, Corrientes 3400, Argentina; 14 Department of Cardiology, Hospital Santo Tomás, Panama City 07093, Panama; 15 Department of Cardiology, Fundación Valle del Lili, Cali 760026, Colombia; 16 Department of Cardiology, Hospital San Vicente de Paúl, Heredia 40101, Costa Rica; 17 Department of Cardiology, Instituto Nacional Cardiovascular, Lima 15072, Peru

**Keywords:** heart failure, COVID-19, intensive care unit, mortality

## Abstract

Since early 2020, different studies have shown an increased prevalence of COVID-19 and poorer prognosis in older adults with cardiovascular comorbidities. This study aimed to assess the impact of heart failure (HF) on cardiovascular complications, intensive care unit (ICU) admissions, and in-hospital mortality in patients hospitalized with COVID-19. The CARDIO COVID 19-20 registry includes 3260 hospitalized patients with a COVID-19 serological diagnosis between May 2020 and June 2021 from Latin American countries. A history of HF was identified in 182 patients (5.6%). In patients with and without previous HF, the incidence of supraventricular arrhythmia was 16.5% vs. 6.3%, respectively (*p* = 0.001), and that of acute coronary syndrome was 7.1% vs. 2.7%, respectively (*p* = 0.001). Patients with a history of HF had higher rates of ICU admission (61.5% vs. 53.1%, respectively; *p* = 0.031) and in-hospital mortality (41.8% vs. 24.5%, respectively; *p* = 0.001) than patients without HF. Cardiovascular mortality at discharge (42.1% vs. 18.5%, respectively; *p* < 0.001) and at 30 days post-discharge (66.7% vs. 18.0%, respectively) was higher for patients with a history of HF than for patients without HF. In patients hospitalized with COVID-19, previous history of HF was associated with a more severe cardiovascular profile, with increased risk of cardiovascular complications, and poor in-hospital and 30-day outcomes.

## 1. Introduction

According to the World Health Organization, SARS-CoV-2 infection (COVID-19) became a pandemic, with over 762 million reported cases worldwide as of April 2023 [1], changing the dynamics of morbidity and mortality worldwide and putting a financial strain on healthcare systems in several countries. Since early 2020, different studies have shown an increased prevalence of COVID-19 and poorer prognosis in older adults with cardiovascular comorbidities [2], with later reports and descriptions affirming these initial findings [3,4,5,6]. Only a few of these focused on analyzing the impact of heart failure (HF) on outcomes after COVID-19 events [7,8], some of which proved that HF is an independent risk factor for mortality [9]. Using the available clinical data collected by 14 Latin American countries from patients hospitalized with COVID-19 and recorded in the CARDIO COVID 19-20 Registry from May 2020 to June 2021, the aim of this study was to evaluate the impact of HF as a comorbidity on intensive care unit (ICU) admissions, the incidence of cardiovascular complications, and in-hospital mortality in patients with COVID-19.

## 2. Materials and Methods

The CARDIO COVID 19-20 Registry sought to determine the presence of comorbidities and cardiovascular complications in patients with COVID-19 who were admitted to Latin American hospitals between May 2020 and June 2021. The registry recruited 3260 adult patients with a COVID-19 diagnosis confirmed using reverse-transcription polymerase chain reaction (RT-PCR) or antigen tests for SARS-CoV-2 at 44 institutions in 14 Latin American countries, including Argentina, Brazil, Chile, Colombia, Costa Rica, Ecuador, El Salvador, Guatemala, Mexico, Panama, Paraguay, Peru, the Dominican Republic, and Venezuela. HF diagnoses, along with information about other comorbidities, were taken from medical histories and clinical records. Cardiovascular complications were documented during hospitalization, including arrhythmia, deep vein thrombosis (DVT), pulmonary embolism (PE), arterial thrombosis, decompensated heart failure (DHF), acute coronary syndrome (for acute coronary syndrome, we classified it as 1. Acute Myocardial Infarction with elevation of ST segment, or 2. Acute Myocardial Infarction without elevation of ST segment), and myocarditis (defined as the evidence of this diagnosis obtained from the clinical records of the recruited patients, and based on the wide range of clinical presentations of this condition, the diagnosis should be differentiated from acute coronary syndrome with atherosclerotic lesions, other causes that can explain abnormal serological biomarkers (pulmonary embolism, acute coronary syndrome, decompensated heart failure, among others), and other conditions explaining new-onset acute decompensated heart failure). Demographic variables such as past medical history of cardiovascular complications, comorbidities, treatment regimens administered during hospitalization, and outcomes were also registered. The full study design, patient recruitment, eligibility criteria, and evaluations were published elsewhere [10]. The Ethics Committee of the Fundación Valle del Lili in Cali, Colombia, approved the registry protocol.

### 2.1. Data Collection

The subject of analysis for this study included 182 patients who had a previous diagnosis of HF (the definition of HF/history of HF was based on the information obtained from clinical records, which included the registered diagnosis of the entity and the evidence in the clinical record of the previous use of specific HF medication for this condition). The Research Electronic Data Capture (REDCap) electronic database system was used to collect information. Demographic information (i.e., age, sex, ethnicity, education, smoking, and pregnancy status), comorbidities before admission (cardiovascular and non-cardiovascular), previous cardiovascular treatment, laboratory test results at admission, clinical findings on admission, cardiovascular complications during hospitalization, echocardiography and other diagnostic cardiovascular tests, cardiovascular procedures, as well as COVID-19 treatments carried out during hospitalization, laboratory test results at discharge, and outcomes both during hospitalization and 30 days after discharge were analyzed.

### 2.2. Main Outcomes

Primary clinical outcomes were ICU admission, in-hospital all-cause mortality, readmission, and 30-day discharge mortality. These variables were documented at the time of discharge. All patients were followed until 30 days after discharge to evaluate rehospitalization or mortality through phone calls.

### 2.3. Statistical Analysis

#### 2.3.1. Univariate and Bivariate Analysis

If the measurements followed a normal distribution, data are reported using mean values and the corresponding standard deviation (SD). If not, medians with a corresponding interquartile range (IQR) are used.

Categorical variables are presented as frequencies and percentages. When data were normally distributed, the independent samples *t*-test was used to compare the means of continuous variables. Otherwise, the Mann–Whitney test was used. Pearson’s Chi-square test and Fisher’s exact test were used to compare proportions between groups. To compare, in terms of mortality, patients who developed DHF and had a history of HF vs. those who developed DHF but had no history of HF, a *t*-test was performed to compare the proportions of the two independent populations. The results were considered statistically significant if the *p*-value < 0.05. All descriptive statistical analysis was performed using the R program (Version 4.2.2).

#### 2.3.2. Multivariate Analysis

The predictor variables of mortality and ICU admission in patients with a history of HF and a diagnosis of COVID-19 were determined using a logistic regression model. The multivariate analysis was completed using Python 3.11.1 (compatible version). For the regression analysis, we filtered the variables analyzed, considering the missing data. If a variable had 20% or more missing data, we did not include it in the analysis. Moreover, we did not include variables that were not clinically relevant.

## 3. Results

A total of 182 out of 3260 recruited patients (5.58%) had a previous diagnosis of HF. The median ages of patients with and without a diagnosis of HF were 71 and 60 years, respectively (Table 1).

### 3.1. Outcomes from Patients with HF vs. without HF History

Comorbidities such as arterial hypertension, diabetes mellitus, dyslipidemia, asthma, chronic obstructive pulmonary disease (COPD), chronic kidney disease (CKD), coronary heart disease, atrial fibrillation (AF), and smoking status were more prevalent (*p* < 0.001) in patients with HF. This group had more radiographic evidence of pulmonary congestion, pleural effusion, and cardiomegaly (42.3%, 22.5%, 53.8% vs. 18.6%, 11%, 16.8%, respectively; *p* < 0.001) (Table 2); lower hemoglobin and hematocrit levels (*p* = 0.001); and higher creatinine, D-dimer, troponin, and NT-proBNP values (*p* = 0.001) at admission (Table 2). These findings were associated with higher myocardial injury most likely associated with COVID-19.

Arrhythmias (22% vs. 8.3%) and acute coronary syndrome (7.1% vs. 2.9%) were the cardiovascular outcomes that showed a statistically significant difference between patients with and without HF, respectively (*p* < 0.001) (Table 3). Furthermore, DHF was diagnosed in 106 patients (58.2%) with a previous diagnosis of HF and only in 171 patients (5.6%) without a previous diagnosis of HF or with de novo HF (*p* = 0.001) (Table 3). No statistical differences in terms of mortality were found when comparing patients who developed DHF and had a history of HF (54 patients; 50.9%) versus patients who developed DHF but had no history of HF (103 patients; 60.2%) (*p* = 0.2784).

Anticoagulants (56.6% vs. 38.6%; *p* < 0.001) and chloroquine (5.5% vs. 2.6%; *p* = 0.037) were the most common treatments administered to patients with a previous diagnosis of HF compared to those without it (Table 4).

Patients with HF also had a higher mortality rate than those without it (41.8% vs. 24.5%, respectively), with non-cardiovascular mortality being more frequent than cardiovascular mortality in both groups (HF: 57.9% vs. 42.1%; *p* < 0.001; without HF: 81.5% vs. 18.5%; *p* < 0.001) (Table 4).

### 3.2. Outcomes from Patients with HF

Table 5 shows the variables associated with increased mortality and ICU admission in patients with HF according to the logistic regression model. Notably, the prediction models used were highly accurate (0.76 and 0.85 for ICU admission and mortality models, respectively). Even though it was not statistically significant, arterial hypertension is included in the table due to its clinical value. HF history was also associated with a higher risk of death (*p* = 0.001) and ICU admission (*p* = 0.031). Inotropic support and invasive mechanical ventilation (IMV) were strongly related to hospital mortality (*p* = 0.027 and *p* = 0.019, respectively).

Mortality and ICU admission according to left ventricular ejection fraction (LVEF) are presented in Table 6.

## 4. Discussion

Our study shows that having HF is associated with increased cardiovascular complications and in-hospital mortality in patients with COVID-19, findings consistent with the reports of other works and from the analyses of large registries [11].

There are multiple articles correlating COVID-19 and cardiovascular disease to worse outcomes and complications [2,3,4,5,6,7,8]. A relationship between COVID-19 infections and cardiac arrhythmias was described by Bhatla et al. [12], with ORs of 4.68 (95% CI 1.66–13.18) for AF and 8.92 (95% CI 1.02–1.09) for non-sustained ventricular tachycardia (NSVT). In our registry, 5.5% of patients with HF experienced ventricular arrhythmias.

Tomasoni et al. [6], in their multicenter registry including 692 patients (Cardio-COVID-Italy), found a HF prevalence of 13%; this population was typically older, and with a higher proportion of cardiovascular risk factors such as arterial hypertension, dyslipidemia, diabetes mellitus, coronary heart disease, and AF, compared to those without HF. There were no reports of supraventricular arrhythmias, and ventricular arrhythmias were present in 5.6% of individuals with HF.

In the CAPACITY COVID Registry, an international registry of 3011 patients, Linschoten et al. [11] reported a 5.3% prevalence of HF, with 349 patients requiring hospitalization for cardiovascular problems. They characterized arrhythmias and conduction disorders as the most frequent cardiac complications, identified in 73.9% of cases, with supraventricular tachycardia accounting for 54.2% of these. Acute coronary syndrome, on the contrary, was only diagnosed in 4.3% of cases, with PE and myocarditis also being uncommon. As per our registry, arterial hypertension, diabetes mellitus, and dyslipidemia were the most prevalent comorbidities in the HF population, being present in 72.5%, 46.2%, and 34.1% of patients, respectively. The findings on ventricular arrhythmias from the trial by Tomasoni et al. [6] are comparable to our study.

Tomasoni et al. [6] also reported higher mortality in individuals with previous HF, a finding differing from that documented by Linschoten et al. [11] but similar to our findings. In a multivariate Cox regression model, the risk of death associated with HF remained elevated after adjusting for clinical variables related to COVID-19 and the severity of the HF such as oxygen saturation, lymphocyte count, and plasma troponin (hazard ratio adjusted for death, 2.25; 95% CI, 1.26–4.02; *p* = 0.006). Interestingly, in our registry, cardiovascular mortality in the general population was 20.5%, compared to 18.5% in patients without HF and 42.1% in patients with HF.

Kim et al. [13] recently reported the findings of the Korean nationwide COVID-19 registry, which included 212,678 patients, and aimed to investigate the relationship between HF and the susceptibility to severe COVID-19 sequelae. The primary composite outcome included IMV, ICU admission, and mortality. Only 310 (4.1%) of the total number of patients had a history of HF. Following propensity score matching, HF comorbidity was revealed to be independently associated with the primary composite outcome (OR: 1.99; 95% CI: 1.42–2.79; *p* < 0.001) and notably with mortality (OR: 2.02; 95% CI: 1.36–3.00; *p* < 0.001). This supports previous published data and is in accordance with our findings [6].

In general, there are several reports associating HF history with a higher risk of complications and a worse prognosis. A study from Spain, including 3080 patients with COVID-19 [14], found that the 152 (4.9%) patients who had a history of HF were more likely to develop acute DHF as a cardiovascular complication during their hospitalization compared to the non-HF group (11.2% vs. 2.1%; *p* < 0.001) and, as a consequence, had a higher mortality risk (46.8% vs. 19.7%; *p* <0.001) [14,15].

Angeli et al. [16] documented a synergistic effect of HF and CAD in 954 consecutive patients hospitalized for SARS-CoV-2 in five Italian hospitals. They found a significant 35.6% mortality rate in the group with HF and CAD (OR: 6.9; *p* < 0.0001). Also described was the risk of mortality in patients with CAD and HF combined, which was consistently higher than the sum of risks related to either disorder. In our study, we found a high prevalence of CAD in the HF population (44.4%). Our analysis did not focus specifically on the relationship of these two comorbidities. However, the high prevalence of CAD in our population could be related to the statistically significant higher mortality rate found in patients with HF vs. patients without HF (41.8% vs. 24.5%, respectively; *p* < 0.001). Also, similar to our study, 44% of the population that Angeli et al. [16] analyzed that had a history of HF also had a history of CAD. Additionally, they cited numerous studies that have evaluated the detrimental prognostic effect of CAD or HF in patients with COVID-19, including monocenter, and multicenter studies from Italy, as well as from Sweden, specifically a registry that showed that CAD is a powerful and independent predictor of death in patients with non-valvular HF. In view of the above, the population in our study that had a history of both CAD and HF could have had higher mortality and, finally, have contributed greatly to the mortality rate found.

Our findings are also consistent with the results of a systematic review and meta-analysis that synthetized evidence from 18 studies worldwide, including 21,640 patients and demonstrating that HF was related to higher rates of hospital admission following COVID-19 infection [17] as well as poorer COVID-19 outcomes (defined by mortality or incidence of severe COVID-19 disease, ICU admission, and requirement of IVM), and a higher mortality rate [15].

In a study published by Raisi-Estabragh et al. [18], based on the UK Biobank, 17,871 patients with COVID-19 were evaluated from the time of infection to up to 30 days of follow-up between March 2020 and 2021. Their analyses revealed that 3% of patients in the COVID-19 group had some form of cardiovascular disease compared to 0.5% in a control group, this condition being more prevalent in individuals who were hospitalized. They also found that they more frequently developed complications such as venous thromboembolism (VTE), atrial fibrillation, and HF.

COVID-19-induced cardiovascular complications are not restricted to the acute phase of the disease, since significant findings have been documented up to one year after infection, even in patients who did not require hospitalization [19].

In 2022, a large-scale study was carried out that included 153,760 patients diagnosed with COVID-19 belonging to the United States Department of Veterans Affairs. This was controlled by two groups: a contemporary cohort of 5,637,647 and a historical cohort (before COVID-19) of 5,859,411 patients, all with a 12-month follow-up [19]. Two main conclusions were drawn from this study: First, even in patients who did not require hospitalization during the acute stage of the disease, the chance of developing cardiovascular problems increased substantially. Second, during the acute phase of the disease, the risk of developing cardiovascular complications rose progressively in direct proportion to the severity of the clinical presentation. The most prevalent cardiovascular complications in this study were supraventricular arrhythmias and HF, demonstrating that the risk of developing the latter was 72% higher in the group that had a history of COVID-19 infection than in the group that did not.

Wang et al. [20] conducted another study, emphasizing the need for a long-term follow-up (up to 12 months) in these patients, after having evaluated the development of cardiovascular complications in patients who survived COVID-19 using data from the US Collaborative Network in the TriNetX database. A total of 4,131,717 individuals with this condition were recruited from a cohort of 42 million adults registered between January 2019 and March 2022. Their analyses concluded that COVID-19 survivors were at an increased risk of the development of cerebrovascular complications such as strokes (HR = 1.52 [1.43 − 1.62]), arrhythmias including atrial fibrillation and flutter (HR = 2407 [2296 − 2523]), myocarditis (HR = 4406 [2890 − 6716]), and HF (HR = 2296 [2200 − 2396]) in the long term.

Those results support the hypothesis that HF is a risk factor for poor outcomes in COVID-19 patients, a problematic situation even in the long term, providing valuable evidence for public health systems and healthcare services in the up-coming years, and hopefully helping to develop adequate strategies for the management of these problems, as they could eventually result in a post-pandemic health system crisis [21].

## 5. Limitations

The limitations of this study can be traced back to the information collection, as some data were incomplete due to the isolation measures in place to contain the pandemic at that time. Additionally, a non-random sampling of the population was conducted, including only medical institutions equipped with hospitalization, ICU, and specific paraclinical facilities as requirements for filling the variables in the registry. However, despite the mentioned limitations, the described findings are valuable and enable an initial appreciation of the clinical course of COVID-19 patients. They should encourage healthcare personnel towards early detection of cardiovascular complications, which are associated with a more severe disease course, increased risk of ICU admission, and mortality.

## 6. Conclusions

In the Latin American population, patients with HF who are hospitalized for COVID-19 have more myocardial injury (changes in troponin, D-dimer, and peptide), increased risk of developing cardiovascular complications, higher ICU admission rates, and higher general and cardiovascular in-hospital mortality rates. At the 30-day follow-up, there was no statistically significant difference in all-cause mortality when compared to the group without HF.

## Figures and Tables

**Table 1:** Demographic characteristics and comorbidities of patients with COVID-19 according to the presence/absence of HF.

Variables	All Patients N = 3260 n (%)	Without HF N = 3078 n (%)	With HF N = 182 n (%)	*p*-Value ^1^
Age (y), median (IQR)	61 (48.0, 71.0)	60 (48.0, 70.0)	71 (60.0, 78.8)	<0.001
Male, n (%)	2059 (63.2)	1944 (63.2)	115 (63.2)	>0.9
Comorbidities				
Arterial hypertension	1596 (49.0)	1464 (47.6)	132 (72.5)	<0.001
Diabetes mellitus	869 (26.7)	785 (25.5)	84 (46.2)	<0.001
Overweight/obesity	1621 (49.7)	1540 (50)	81 (44.5)	0.2
Coronary heart disease	244 (7.5)	163 (5.3)	81 (44.4)	<0.001
Atrial fibrillation	115 (3.5)	65 (2.1)	50 (27.5)	<0.001
Dyslipidemia	451 (13.8)	389 (12.6)	62 (34.1)	<0.001
Chronic lung disease/asthma	287 (8.8)	256 (8.3)	31 (17)	<0.001
Chronic kidney disease	270 (8.3)	223 (7.3)	47 (25.7)	<0.001
Cancer	139 (4.3)	127 (4.1)	12 (6.6)	0.2
Cerebrovascular event	102 (3.1)	92 (3.0)	10 (5.5)	0.10
HIV infection	24 (0.7)	24 (0.8)	0 (0.0)	0.5
Smoking	438 (13.4)	386 (12.5)	52 (28.6)	<0.001

^1^ Wilcoxon rank sum test; Pearson’s Chi-squared test. HF: heart failure. HIV: human immunodeficiency virus. IQR: interquartile range. n: number of patients. y: years.

**Table 2:** Clinical and paraclinical characteristics of patients with COVID-19 according to the presence/absence of HF.

Variables	All Patients N = 3260 n (%)	without HF N = 3078 n (%)	with HF N = 182 n (%)	*p*-Value ^1^
Clinical manifestations				
Fever	2099 (64.4)	2022 (65.7)	77 (42.3)	<0.001
Cough	2235 (68.6)	2135 (69.4)	100 (54.9)	<0.001
Fatigue	1625 (49.8)	1528 (49.1)	97 (53.3)	0.4
Anorexia	634 (19.4)	594 (19.3)	40 (22.0)	0.4
Myalgias	1101 (33.8)	1071 (34.8)	30 (16.5)	<0.001
Diarrhea	456 (14.0)	443 (14.4)	13 (7.1)	0.009
Chest pain	446 (13.7)	412 (13.4)	34 (18.7)	0.056
Palpitations	151 (4.6)	127 (4.1)	24 (13.2)	<0.001
Dyspnea	2365 (72.5)	2228 (72.4)	137 (75.3)	0.4
Loss of taste	213 (6.5)	208 (6.8)	5 (2.7)	0.048
Loss of smell	223 (6.8)	219 (7.1)	4 (2.2)	0.016
Other	1132 (34.7)	1085 (35.3)	47 (25.8)	0.012
Laboratories at admission, median (IQR)				
Hemoglobin, mg/dL	13.6 (12.1, 14.9)	13.7 (12.2, 14.9)	12.5 (10.7, 14.0)	<0.001
Hematocrit, %	40.2 (36.1, 44.0)	40.4 (36.4, 44.0)	37.6 (31.7, 42.3)	<0.001
Leukocytes, ×10^3^ μL	8.7 (6.3, 12.1)	8.7 (6.3, 12.2)	8.5 (6.4, 11.6)	0.5
Platelets, ×10^3^ μL	228 (176, 297)	230 (177, 299)	210 (160, 267)	<0.001
Creatinine, mg/dL	0.9 (0.7, 1.2)	0.9 (0.7, 1.2)	1.2 (0.8, 2.0)	<0.001
Ureic nitrogen, mg/dL	18.0 (12.5, 28.9)	17.5 (12.3, 27.3)	29.0 (20.0, 51.8)	<0.001
Aspartate Transaminase, U/L	42 (29.0, 65.7)	42.1 (29.0, 66.0)	36.0 (25.0, 63.8)	0.12
Alanine aminotransferase, U/L	37 (24.0, 60.0)	37.9 (24.3, 61.0)	31.0 (18.1, 49.2)	0.002
D-dimer, mcg/mL	0.75 (0.38, 1.53)	0.73 (0.37, 1.52)	0.94 (0.51, 2.38)	0.003
Ferritin, ng/mL	820 (396, 1481)	831 (398, 1491)	691 (377, 1272)	0.063
Ultrasensitive troponin I, ng/mL	0.01 (0.01, 0.03)	0.01 (0.01, 0.02)	0.02 (0.01, 0.11)	<0.001
Lactic dehydrogenase, U/L	369 (269, 515)	369 (269, 512)	380 (264, 543)	0.6
NT-proBNP, pg/mL	429 (88, 2744)	312 (81, 1932)	3357 (2140, 9132)	<0.001
Chest x-ray				
Pulmonary infiltrates	2605 (79.9)	2478 (80.5)	127 (69.8)	<0.001
Pulmonary congestion	608 (18.6)	531 (17.3)	77 (42.3)	<0.001
Pleural effusion	360 (11)	319 (10.4)	41 (22.5)	<0.001
Cardiomegaly	549 (16.8)	451 (14.7)	98 (53.8)	<0.001

^1^ Wilcoxon rank sum test; Pearson’s Chi-squared test. HF: heart failure. IQR: interquartile range. n: number of patients. NT-proBNP: N-terminal (NT)-prohormone brain natriuretic peptide.

**Table 3:** Cardiovascular complications during hospitalization.

Variables	All Patients N = 3260 n (%)	Without HF N = 3078 n (%)	With HF N = 182 n (%)	*p*-Value ^1^
Arrhythmias	296 (9.1)	256 (8.3)	40 (22.0)	<0.001
Supraventricular	224 (6.9)	194 (6.3)	30 (16.5)	<0.001
Ventricular fibrillation	72 (2.2)	62 (2.0)	10 (5.5)	0.2
DVT	40 (1.2)	40 (1.3)	0 (0.0)	0.2
PE	126 (3.9)	118 (3.8)	8 (4.4)	0.9
Arterial thrombosis	21 (0.6)	20 (0.6)	1 (0.5)	>0.9
Decompensated HF	277 (8.5)	171 (5.6)	106 (58.2)	<0.001
Congestion	176 (5.4)	104 (3.4)	72 (39.6)	<0.001
High blood pressure	10 (0.3)	7 (0.2)	3 (1.6)	0.2
Pulmonary edema	34 (1.0)	24 (0.8)	10 (5.5)	0.10
Cardiogenic shock	58 (1.8)	37 (1.2)	21 (11.5)	0.5
Acute coronary syndrome	94 (2.9)	81 (2.7)	13 (7.1)	<0.001
Myocarditis	40 (1.2)	35 (1.1)	5 (2.7)	0.12

^1^ Wilcoxon rank sum test; Pearson’s Chi-squared test. DVT: deep vein thrombosis. HF: heart failure. PE: pulmonary embolism.

**Table 4:** Treatment for COVID-19 and outcomes.

Variables	All Patients N = 3260 n (%)	Without HF N = 3078 n (%)	With HF N = 182 n (%)	*p*-Value ^1^
COVID-19 treatment, n (%), median (IQR)				
Corticosteroids	2197 (67.3)	2087 (67.8)	110 (60.4)	0.12
Azithromycin	1096 (33.6)	1049 (34.1)	47 (25.8)	0.027
Hydroxychloroquine	690 (21.2)	669 (21.7)	21 (11.5)	0.001
Chloroquine	90 (2.8)	80 (2.6)	10 (5.5)	0.037
Lopinavir	234 (7.2)	228 (7.4)	6 (3.3)	0.052
Ritonavir	229 (7.0)	223 (7.2)	6 (3.3)	0.061
Interferon	9 (0.3)	9 (0.3)	0 (0.0)	>0.9
Immunoglobulins	23 (0.7)	21 (0.7)	2 (1.1)	0.8
Anticoagulants	1257 (38.6)	1154 (37.5)	103 (56.6)	<0.001
Thromboprophylaxis	2021 (62.0)	1928 (62.6)	93 (51.1)	0.002
Plasmapheresis	31 (1.0)	29 (0.9)	2 (1.1)	>0.9
Severity and outcome variables, n (%), median (IQR)				
Vasopressors	900 (27.6)	837 (27.2)	63 (34.6)	0.037
Vasopressor days	7.0 (3.0, 12.8)	7.0 (3.0, 13.0)	6.5 (3.0, 12.0)	0.8
Inotropic	336 (10.3)	286 (9.3)	50 (27.5)	<0.001
Inotropic days	5.0 (3.0, 10.0)	5.0 (3.0, 9.0)	6.0 (4.0, 10.8)	0.086
ICU hospitalization	1745 (53.5)	1633 (53.1)	112 (61.5)	0.031
ICU days	10.0 (5.0, 18.0)	10.0 (5.0, 18.0)	10.0 (4.8, 18.0)	0.8
IMV Requirement	1115 (34.2)	1054 (34.2)	61 (33.5)	>0.9
IMV days	11.0 (6.0, 19.0)	11.0 (6.0, 19.0)	8.0 (4.0, 20.0)	0.4
Mortality	831 (25.5)	755 (24.5)	76 (41.8)	<0.001
Cardiovascular	172 (20.7)	140 (18.5)	32 (42.1)	<0.001
Non-cardiovascular	659 (79.3)	615 (81.5)	44 (57.9)	
Follow-up 30 days post-discharge *n* = 2427				
Rehospitalization, n (%)	144 (7.3)	134 (7.1)	10 (11.5)	0.2
No data		433	19	
Mortality, n (%)	53 (2.6)	50 (2.6)	3 (3.3)	0.2
Cardiovascular	11 (20.8)	9 (18.0)	2 (66.7)	
Non-cardiovascular	42 (79.2)	41 (82.0)	1 (33.3)	

^1^ Wilcoxon rank sum test; Pearson’s Chi-squared test. HF: heart failure. ICU: intensive care unit. IMV: invasive mechanical ventilation. IQR: interquartile range. n: number of patients.

**Table 5:** Variables associated with mortality and ICU admission in patients with HF according to the logistic regression model.

Variable	Coef	OR	95% CI	*p*-Value
Mortality				
Leukocytes at admission	1.363	3.908	[1.802–8.475]	0.001
Lymphocytes at admission	−0.859	0.424	[0.252–0.712]	0.001
Hemoglobin at admission	−0.585	0.557	[0.353–0.879]	0.012
Platelet count at admission	−0.714	0.490	[0.295–0.814]	0.006
Blood urea nitrogen at admission	0.966	2.628	[1.525–4.526]	0.000
High blood pressure	0.920	2.509	[0.848–7.419]	0.096
Use of inotropes	1.225	3.404	[1.146–10.116]	0.027
IMV	1.154	3.171	[1.209–8.317]	0.019
ICU admission				
Aspartate transaminase	2.139	8.493	[1.048–68.853]	0.045
Hemoglobin at admission	−0.541	0.582	[0.392–0.866]	0.008
ACE inhibitors	1.787	5.971	[2.405–14.824]	0.000
No cardiac arrhythmia	−1.336	0.263	[0.093–0.741]	0.011
Fever	0.637	1.890	[0.876–4.077]	0.105
Central venous catheter	1.996	7.357	[2.481–21.819]	0.000
Hydroxychloroquine	2.375	10.752	[2.171–53.254]	0.004

ACE: angiotensin-converting enzyme. CI: confidence interval. ICU: intensive care unit. IMV: invasive mechanical ventilation. OR: odds ratio.

**Table 6:** Mortality and ICU admission percentages of patients with HF according to LVEF values.

LVEF	N	ICU Admissions n (%)	Mortality n (%)
<40%	72	43 (59.7)	31 (43.1)
40–50%	36	22 (61.1)	14 (38.9)
>50%	42	31 (73.8)	14 (33.3)

ICU: intensive care unit. n: number of patients. LVEF: left ventricular ejection fraction.

## Data Availability

The data that support the findings of this study are available from the corresponding author upon reasonable request.
